# Globalization and Health: developing the journal to advance the field

**DOI:** 10.1186/s12992-016-0143-2

**Published:** 2016-03-09

**Authors:** Greg Martin, Malcolm MacLachlan, Ronald Labonté, Fiona Larkan, Frédérique Vallières, Niamh Bergin

**Affiliations:** Centre for Global Health, Trinity College, University of Dublin, Dublin, Ireland; School of Psychology, Trinity College, University of Dublin, Dublin, Ireland; Centre for Rehabilitation Studies, Stellenbosch University, Stellenbosch, South Africa; Palacky University, Olomouc, Czech Republic; School of Epidemiology, Public Health and Preventive Medicine, University of Ottawa, Ottawa, Canada; School of Medicine, Trinity College, University of Dublin, Dublin, Ireland; Masters in Global Mental Health Programme, University of Glasgow, Glasgow, Scotland

## Abstract

Founded in 2005, *Globalization and Health* was the first open access global health journal. The journal has since expanded the field, and its influence, with the number of downloaded papers rising 17-fold, to over 4 million. Its ground-breaking papers, leading authors -including a Nobel Prize winner- and an impact factor of 2.25 place it among the top global health journals in the world. To mark the ten years since the journal’s founding, we, members of the current editorial board, undertook a review of the journal’s progress over the last decade. Through the application of an inductive thematic analysis, we systematically identified themes of research published in the journal from 2005 to 2014. We identify key areas the journal has promoted and consider these in the context of an existing framework, identify current gaps in global health research and highlight areas we, as a journal, would like to see strengthened.

## Background

Published in 2005 as the first open-access global health journal, *Globalization and Health* offers an international platform for quality original research, knowledge sharing, and debate on the topic of globalisation and its effects on health. The journal assumes a cross-sector and multidisciplinary approach, inviting scholarship from clinical, biological, social, political, economic, environmental and information sciences. It caters to a wide audience including: academics, policy-makers, health care practitioners, and public health professionals. After a decade of publishing, the journal has become a trusted source of high quality peer reviewed papers.

As of 2015, article publications have more than quadrupled from 18 papers published in 2005, to 81 papers in 2014 and we currently publish more than a third of all submissions. Over 4 million papers have been downloaded, resulting in more than 2300 individual citations. We continue to attract authors from across the world and from diverse backgrounds, including academics, policy makers, humanitarian and development aid workers, Ministers of Health, students and a Nobel Prize winner. *Globalization and Health* is rapidly climbing the ranks of public health related journals in the world, and our impact factor rising from 1.485 in 2012 to 2.25 in 2014. We are particularly proud of the international reach of our papers and of our free-to-publish provision for authors from low-income countries.

The journal has covered the major global health events of the last fifteen years, including the SARS virus outbreak of the early 2000s, the politics of the tobacco industry, the ‘Westernization’ of lifestyle behaviours and its associated health implications, access to essential medicines, and the most recent Ebola crisis in West Africa: all from the perspective both of high-income and low and middle-income countries. To mark our tenth year as a journal, and the migration of the journal to its new institutional address at the Centre for Global Health, Trinity College Dublin, members of the current editorial board undertook a review of all the articles that have been published to date. The purpose of this review was to identify strengths, trends and current gaps in research; highlight opportunities for strengthening existing research; and to propose upcoming areas of research, projected to be of increasing importance over the next ten years.

## Methodology

**Fig. 1:**
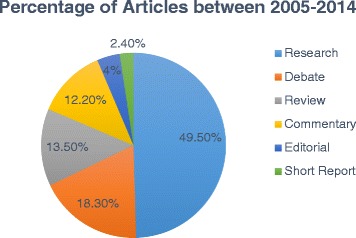
Percentage of Journal Articles Published by Type between 2005 and 2014

An analysis was undertaken of all articles published between the years 2005 and 2014 (*n* = 327), using a three-stage inductive thematic approach. In the first stage, an open coding process was applied, whereby all abstracts were read and 43 overall themes identified. In the second stage, these 43 themes were reassigned to 20 thematic categories with the help of a second researcher (Table 2, Column A). Subthemes were then identified through rereading of abstracts, and the paper in full in cases where it was felt that subthemes were not obvious from the abstract (Table 2, Column B). Finally, axial coding was used to identify similarities or overlaps in thematic categories and across papers in the context of existing frameworks in global health research [1, 2] (see Table 3). As an interim step, descriptive characteristics were drawn from each paper, including the number and country of origin of authors (Table 1), type of journal article (Fig. 1), regions of focus of the paper, and disciplinary focus. These are presented below.

**Table 1:** Location of primary author by continent

Continent	Percentage of first authors	No. of papers
North America	37 %	121
Europe	35.8 %	117
Africa	10.7 %	35
Asia	7.3 %	24
Australia/Oceania	7 %	23
South America	2.1 %	7

## Results

### Descriptives

Table 1 summarises the location of the affiliation of the first author and indicates that the majority of authors came from northern hemisphere, high-income countries. The USA accounted for 24 % of all authors, while the UK made up 12 %. North America and Europe shared an equally high percentage of primary authors (36 % and 35 %, respectively).

First authors most frequently identified themselves with Medicine and Public Health disciplines (15 %). Multi-disciplinary backgrounds (32 articles (9.8 %)) and undisclosed disciplines (45 articles (13.8 %)) were also frequent. Global Health, Epidemiology, Health Science and Population Health were among the most commonly identified disciplines. Figure 1 summarises the six types of journal articles published:

In the early years of the journal there was no clear preference for research articles (2005–2007). However from 2011 onwards, research articles accounted for over 50 % of the publications in the journal. Literature reviews were also favoured (9.5 %). Approximately 17 % of papers were classed as highly accessed by the journal’s publisher, Bio-Med Central (BMC).

The region of focus was defined as the geographical area where the research took place, or the area that was a primary concern of the research. Regions were either generalised (e.g. worldwide) or specific (e.g. Rwanda). A generalised view was the most common (30 %), with 55 articles (16.8 %) focused specifically on low and middle-income countries (LMICs). This reflects the high number of meta-analysis and review articles that used data from across countries, rather than from a single country. Eight articles (2.4 %) examined or contrasted between low-income and high-income countries. Canada was the most frequently cited country (2.4 %), with research primarily focused on the reasons for, and policies surrounding, medical tourism [3, 4].

### Key thematic categories

Table 2, Column A presents the 20 key thematic categories identified from the analysis, as described above. The first six of these (health systems, pharmaceuticals, communicable diseases, non-communicable diseases, research, policy-making and migration) were considered particularly ‘rich’ as they contained six or more subthemes. A second tier of thematic categories, each with 3 or 4 sub-themes included health technologies, international aid, and global health as an area of study, partnerships and knowledge sharing, nutrition, mental health and global threats. A third set of thematic categories although less ‘rich’ in terms of the range of sub-themes, included many areas critical for global health, such as tobacco control, maternal health and the health and rights of sex workers.

**Table 2:** Analysis of thematic categories and sub-themes published in *Globalization and Health* 2005-2015

(A) Thematic categories	(B) Sub-themes
Health systems	Accreditation
	Private health sector
	Public health sector
	Economic impact
	Reform of systems for localisation
	Patient care and safety
	Resources
	Access to healthcare
	Education
Pharmaceuticals	Cost of medicines
	Access to medicine
	Local production of medicine
	Implications of drug patents
	Antibiotics
	Antimicrobial resistance
	Governance in the pharmaceutical sector
	Bilateral trade
	Drug trials
Non-communicable diseases	Economic and health burden
	Prevention and intervention
	Diabetes
	Policy making around NCDs
	Home based vs community based care for NCDs
	Socio-economic status
	Cardio vascular disease
	Diabetes and nutrition
Communicable diseases	HIV and AIDS
	ART- sustained delivery and adherence
	Programme sustainability
	Community based awareness and prevention
	Health rights of HIV patients
	Healthcare equity
	AIDS free generation and HIV/AIDS as a chronic illness
Research	Contrasting global north and south
	Drug development
	Funding
	Open access publishing
	Conducting research on diseases
	Scientific rigour
	Underreporting in low and middle income countries.
Policy making – national & global levels	Home based care
	Implementation of policies
	Incentives to improve
	Spending on health
	Millennium development goals and future policy
	Development
	Global fund
Migration	Obesity
	Sexual violence against migrants-prevention
	Migrant health access
	Refugees and NCDs
	Brain drain
	Therapeutic relationships
Technology	eHealth governance and legislation
	mHealth and NCDs
	mHealth and mental health
	Standardisation in health technology
	Telemedicine
International aid	Foreign aid
	Volunteering
	Ethical and sustainable volunteering
	Coordination of aid
	Satisfaction and effectiveness (with usage)
Global health as a field of study	Contributions to global health
	Governance of global health
	Evolution of the field
	Priorities
Partnerships & knowledge sharing	Reverse partnerships in volunteering
	International cooperation
	‘Reverse innovation’
	Open innovation in low resource settings
Nutrition	Obesity in children
	Nutrition and chronic disease
	Global response to obesity
	Implications of trade agreements on nutrition
Mental health	Suicide and socio-economic status
	Work stress, ageing and depression
	Human rights
	Psychological impact of caregiving
Global threats	Weapon control
	Nutrition issues
	Organised crime
	Climate change
Tobacco	Tobacco control
	Nicotine replacement
	Health and economic burden of consumption
Maternal health	Maternal mental health
	Mother to child HIV transmission
	Infection control and maternal mortality
Trade	Pesticide residue on imported food
	TRIPS
	Trade related diseases
Sustainable development	Sustainable impact of volunteerism
	Nutrition
	Sustainable water sanitation
Sex workers	Health interventions
	Alcohol use and HIV vulnerability
	Human rights violations
Global disease	NCDs
	Multi-morbidity
	Epidemiology

Whilst the above analysis is useful insofar as it maps the broad range of topics published by the journal*,* we further reviewed these thematic categories in the context of an existing framework for globalisation and health. Table 3 therefore reinterprets the above thematic categories and subthemes in terms of Labonte and Torgerson’s framework [2] with a view to highlighting existing gaps and informing future research. Table 3 is followed by detailed description of eight key constructs (adapted from Labonte and Torgerson’s framework), and discussed in terms of papers published in *Globalization and Health* over the last ten years.

**Table 3:** Thematic categories and subthemes aligned to key constructs of Labonte and Torgerson’s [2] conceptual framework

Key constructs (Labonte & Torgerson)	Thematic categories	Sub-themes	Examples from G&H
Global policy space & global health contexts	Global policies	Weapon control	[45]-Globalising weapon trade
	Global health	Epidemiology	[46]-Accountability in global health cooperation
	Governance in health and pharmaceuticals	Food globalisation	[47]- Governments aiding nutrition crisis
	Accountability	Global fund	
	Nutrition	Millennium	
		Development goals	
Global & domestic development contexts	Scientific research	Brain drain	[48] - Open access publishing in LMIC
	Educational development	Open innovation	[49]- Reverse innovation and volunteering
	Global partnerships	Disease prevention strategies	[50]- Sustainable development in health
	Development issues in LMIC	Resource allocation and effectiveness,	[51]- Coordination of development assistance
	Voluntourism aid and NGOs	Emerging economies	
	Sustainable development	Open publishing	
		Underreporting in developing countries.	
Environmental pathways	Climate change	Impact on spread of diseases	[52]- Climate change and mosquito borne illnesses
		Climate sensitive health investment	[53]- Global health adaption with climate change
Trade agreements & regulatory space of pharmaceutical products	Human rights	Antimicrobial resistance	[54]- Policy and access to medicine
	Implications of drug patents	Local production of medication	[39]- Import and production of generic medicine
	Cost of medicines	Bilateral trade	
	Access to medicine		
Healthcare systems	Healthcare regulation	Health rights	[55]- Conceptual framework for medical tourism
	Technology and health	Discourse on disease	[28]- eHealth legislation
	Medical tourism	Telemedicine e/mHealth	[56]- Health market regulation in LMIC
	Healthcare systems in LMIC	Access to healthcare systems	[57]- Access to healthcare in post conflict settings
	Maternal health	Water sanitation	[58] - Facilitating access to healthcare
	Public health service regulations	Mother to child HIV	
	Female sex workers’ access to healthcare	Maternal mental health	
Domestic policy & national level contexts/influences	Governmental spending on health	Health system frameworks	[59]- Economic impact of spending on health
	Policy Global health diplomacy	Reducing practice-implementation gap	[60]- Health 'quality chasm’ in resource limited settings
		Policy making on care systems	[32]- Frameworks learning from other international experience
		Improvement incentives	
Population level health influences: NCDs	Prevention and intervention	Prevalence	[61]- Effects of diabetes on domestic health system
	Burden- economic and health	Care systems	[62]- Integrating mHealth and mental health care
	Diabetes	Policy management	[63]- Nicotine replacement therapies
	Cardio vascular disease	Psychological impacts	[64]- Framework for prevention and control of NCDs
	Tobacco	Mental health and SES Suicide	
	Mental health	Tobacco’s global mortality	
	Nutrition	Nicotine replacement and control	
	Alcohol abuse	Obesity nutrition crisis	
Population level health influences: communicable diseases	Transmission of diseases	Adherence and sustained delivery of ART	[65]- Inequity in HIV care
	HIV/AIDS Programme sustainability	Access and supply of interventions	[66]- Access barriers to HIV/AIDS services
	Awareness and prevention ARTs	AIDS free generation	[67]- Psychological influences of AIDS as chronic illness
		Barriers in service	
		Disease reconfiguration- HIV/AIDs as a chronic disease	

#### Global policy space and global health contexts

Includes papers that discuss issues that influence, and/or relate to, health at an international level; specifically, global trade policy initiatives and their impact on health outcomes. Examples include papers related to nutrition and the globalisation of disease due to the global trade of processed food, and their health impact on global obesity [5, 6], as well as trade, globalization processes and the rise in non-communicable disease rates [7]. Papers on international health policies (i.e. papers pertaining to the Global Fund and the Millennium Development Goals [8, 9]) were also categorised within this theme, as was the progression of the field of global health and its evolving definition [10, 11].

#### Global and domestic development contexts

Development was at the core of this key construct, with sustainable development in relation to aid, volunteering policies, emerging pathways of development for LMICs, global health partnerships and scientific research all being discussed. Poverty reduction strategy papers, as mentioned within Labonte and Torgerson’s framework [2], were evident, with a focus on the conceptualisation of aid [12] and on alignment of spending and domestic priorities [13]. The development of research was a topic within this; authors focused upon under-representation of low-income countries in the literature, research partnerships and ethical standards of research [14–16]. Sustainability of global and local initiatives were also covered [17], as was the concept of ‘reverse innovation’ [18].

#### Environmental pathways

Focused primarily upon the impact of globalisation on climate change, and how climate change in turn impacts on health outcomes. Research topics explored climate-sensitive health investments [19] and sustainable community level interventions [20], both aligned to the environmental protection policy level in Labonte and Torgerson’s framework [2].

#### Trade agreements and regulatory space of pharmaceutical products

The pharmaceutical industry, its trade and access to medicines were the research topics in this construct. Authors reported on drug patents and generic manufacturing [21], localised and multinational pharmaceutical companies’ production of medicine [22, 23], and access to medicine [24]. Discussions on these issues were at times framed in relation to intellectual property rights and human rights [25, 26]. Articles on intellectual property rights, drug costs and patent terms dominated topics related to trade.

#### Healthcare systems

Refers to the regulation, organisation, service availability and delivery of health systems. This construct is similar to the ‘health care system’ aspect of Woodward and colleagues’ 2001 framework [1] and includes research papers on technological (i.e. mobile health) and health systems [27] and the legislation around this emerging field [28, 29]. The place of female sex workers within health systems was also included under this construct [30].

#### Domestic policy and national level influences

Firstly refers to the policies made and implemented at a domestic level, for example, the place of community home-based care within national policies [31]. Second, this construct refers to issues which influence policy-making, such as governmental agendas and adequate health system performance (institutional preparedness to support policies which are implemented) [32].

#### Population level influences - NCDs

This construct incorporated research on NCDs and their burden in different contexts (e.g. economic and health burden) and pathways to care (home vs. community care, intervention and prevention). Diabetes was frequently mentioned, both alone and in conjunction with articles on other NCDs, with diabetes being the most commonly cited NCD. Articles focused on interventions for diabetes and recommendations for new or improved options for the prevention and treatment of diabetes. NCDs often arose within other constructs, such as globalised trade [7, 33]. The burden of NCDs, on the economy, government, and people, were also investigated.

#### Population level influences - communicable disease

This construct refers to the influence of communicable disease on the health of a population and both community-based and domestic-level interventions to address these. Research focused on the transmission of communicable diseases, and the influence that globalisation has on the spread of these [34, 35]. Most papers were in relation to HIV and AIDS, and included HIV programme sustainability, access to interventions and adherence to treatments [17, 36, 37]. Sustained use and access to antiretroviral therapy was another frequently occurring topic under this construct. The changing discourse surrounding HIV and AIDS from an incurable disease to a chronic illness was also investigated and the policies, both global and at national level, were explored to see if they were reflective of this change [38]. In this regard, HIV was also closely linked with the NCDs theme, as more papers emphasised HIV’s chronicity. HIV and ART were predominantly discussed in the context of Africa and through prevention, intervention and treatments.

The inextricability of many of the above constructs lends itself to overlap between thematic categories. For example, policy was a commonly occurring topic and many issues were analysed and discussed in the context of their wider policies (e.g. policy on drug patents) [39]. There was also a focus on the extent to which global efforts, such as the Millennium Development Goals, were being achieved and how they might be altered to render them more effective [8].

### Political inclinations of the article

Articles classed as political were those concerned with policy and policy making. These ranged from topics of the policy around home-based care [31] to the globalisation of crime [40]. In a supplementary analysis, articles were marked as being political (30 %) when they explicitly referenced national or global political action. However, it must be noted that it was at times difficult to disaggregate different levels of political engagement, so the classification of articles as political may not be as robust as the other categories in the analysis, with 5 % of the articles being impossible to classify.

- Not political 65 %
- Political 30 %

### A distinctive ethos and direction for future research

Many of the themes identified in this research fit with Labonte and Torgerson’s [2] assertion that research in global health must go beyond a disease specific focus and come to include the social, environmental and economic contexts in which disease occurs. So while many of the principal themes identified were diseases - including HIV and AIDS, diabetes and other NCDs – they were often considered through broader situational and contextual factors, whether community, national, or global. We believe that the interplay between local contexts and global factors influencing health is a particularly valuable and distinctive contribution of *Globalization and Health*. Much of the research explored the national and community contexts in which diseases occurred, with papers focusing upon how strategic plans can be developed at national level to tackle the burden of NCDs [41]. Papers also highlighted the importance of disseminating evidence from local research on health outcomes to national and global levels [42, 43].

The challenges faced by the international development and public health communities are evolving. In light of increasing pressure on the environment, emerging and protracted conflict, political and economic instability, novel zoonotic pandemics, the role of multinationals in global health, and the threat of bioterrorism, future research must be met with timely and evidence-informed responses emerging from innovative technologies, new and broadly stated Sustainable Development Goals, the creation of more effective models of global governance for health, and on-going discussion platforms, such as *Globalization and Health*. Table 4 suggests areas where the journal could respond to these increasing demands, and in so doing continue to reflect, report and influence the complex and compelling interplay between globalisation and health.

**Table 4:** Suggestions for future themes within *Globalization and Health*, reflecting the anticipated development of the field over the next decade

Suggested future themes within *Globalization and Health*	Rationale
Mental health	An important emerging field in global health discourse. The journal has had five publications thus far with mental health as the primary research topic. While mental health may be covered under the general term ‘NCD’ its focus within ‘Globalization and Health’ has not been as substantial as other NCD areas.
Human resources	The dearth of human resources for health continues to act as one of the most important barriers to achieving health for all. As both the HIV and Ebola epidemics demonstrated, the absence of trained health workers, especially front-line health workers, exacerbate the spread of epidemics. This migration of health personnel, mostly from poor countries to rich countries is facilitated by an increasingly globalised world.
Health technology	Advancements in technology (e.g. eHealth, mHealth, telemedicine, assistive products and medical devices) have created immense promise for a more efficient and inclusive delivery of health care. To be effective however, technology must also be accompanied by a capable and motivated user, and an effective system of support and maintenance, where appropriate.
Gender, equity and human rights	Human rights, including the ‘right to health’, have not been prominent in the journal, with only 2 publications coded as having human rights as a main theme. This is somewhat surprising given that the WHO Constitution ‘enshrines the highest attainable standard of health as a fundamental right of every human being’. This theme includes global health law and treaties that impact on human rights.
Migration	Issues surrounding migration, such as the brain drain and sexual violence against migrants have featured thus far in the journal. Health access and issues faced by migrants have also been explored. Climate change and natural resource depletion are expected to increasingly drive migration, both of which have inherently global causes and consequences.
Sustainable development goals	Research in the journal thus far has reviewed health systems in relation to the MDG, where papers have highlighted the top down approach taken to their establishment and the difficulties in implementing them in the global south [8, 68]. The SDGs present a new opportunity to encourage research with a different emphasis, particularly on coherence (or incoherence) between the different goals, their measurement, government accountability for compliance and global financing for the SDGs.
Intercultural aspects of global health	The journal has yet to include much on pluralist health environments and the complexities that such environments pose for practitioners. The role of traditional health knowledge, the role of traditional health practices and practitioners, cultural and communicative competency in delivering international health programs, protection of cultural health knowledge, differential health risks of indigenous populations.
Transnational corporations and health	The size and reach of transnational corporations has been one of the dominant features of contemporary globalisation. Health benefits via economic growth and employment are offset by the diffusion of hazardous products and the environmental and social damages associated with extractive industries. Attempts to regulate their practices have been countered by claims of voluntary corporate social responsibility. Some attention to these issues has been given in this journal, but more is needed as the global health influence of these corporations continues to rise.
Health and global security	A dominating concern in global health is that of health security, reducing the risk of novel pathogens and the rise and spread of antimicrobial resistant diseases. The journal has paid some attention to this aspect of health and global security. But health is also affected by other security issues, ranging from regional conflicts and their causes, the ‘war on terror’, and the health opportunity costs of militarization. Health has also been mooted as a ‘peace dividend’ in conflict areas, while international health work in conflict areas or fragile states poses particular challenges.

## Conclusion

Over the last ten years, *Globalization and Health* has become a trusted source of peer reviewed research and discussion. Over the next ten years, we will continue to facilitate research dissemination and encourage debate by engaging authors and their audiences to suggest evidence-informed and ethically-grounded global health policy and programmatic action. In a rapidly changing global health landscape, we aim to increase the interdisciplinary nature of global health through increased participation from research on cultural perspectives, climate science, mathematical modelling, behavioural sciences, anthropology, international law, big data, history, agricultural science, business science, public policy and administration, and political science. As well as promoting these areas of content we also want to encourage submissions that address the context and process of global health interventions [44]. With a refreshed editorial Board in place for 2016, we thank those who have contributed as editors, reviewers and authors over the past decade; and those who have enhanced or initiated their involvement for the years ahead. We are always open to new ideas, proposals for special issues and collaborations, and innovative suggestions - from anyone - for how we can best reflect and influence globalisation and health.

## References

[1] Woodward D, Drager N (2001). Globalization and health: a framework for analysis and action. Bull World Health Organ.

[2] Labonte R, Torgerson R (2005). Interrogating globalization, health and development: towards a comprehensive framework for research, policy and political action. Crit Public Health.

[3] Turner L, Siegrist J, Lunau T, Wahrendorf M (2012). Beyond “medical tourism”: Canadian companies marketing medical travel. Glob Health.

[4] Johnston R, Crooks VA, Snyder J (2012). “I didn’t even know what I was looking for”: a qualitative study of the decision-making processes of Canadian medical tourists. Glob Health.

[5] Rayner G, Gracia M, Young E, Mauleon JR, Luque E, Rivera-Ferre MG (2010). Why are we fat? Discussions on the socioeconomic dimensions and responses to obesity. Glob Health.

[6] Snowdon W, Raj A, Reeve E, Guerrero RL, Fesaitu J, Cateine K (2013). Processed foods available in the Pacific Islands. Glob Health.

[7] Labonte R, Mohindra KS, Lencucha R (2011). Framing international trade and chronic disease. Glob Health.

[8] Ooms G, Stuckler D, Basu S, McKee M (2010). Financing the millennium development goals for health and beyond: sustaining the “Big Push”. Glob Health.

[9] Gómez EJ, Atun R (2012). The effects of global fund financing on health governance in Brazil. Glob Health.

[10] Rowson M, Willott C, Hughes R, Maini A, Martin S (2012). Conceptualising global health: theoretical issues and their relevance for teaching. Glob Health.

[11] Bozorgmehr K (2010). Rethinking the “global” in global health: a dialectic approach. Glob Health.

[12] Ooms G, Hammonds R, Waris A, Criel B, Van Damme W, Whiteside A (2014). Beyond health aid: would an international equalization scheme for universal health coverage serve the international collective interest?. Glob Health.

[13] Stierman E, Ssengooba F, Bennett S (2013). Aid alignment: a longer term lens on trends in development assistance for health in Uganda. Glob Health.

[14] Pappas G, Hyder AA (2005). Exploring ethical considerations for the use of biological and physiological markers in population-based surveys in less developed countries. Glob Health.

[15] Aikins AD-G, Arhinful DK, Pitchforth E, Ogedegbe G, Allotey P, Agyemang C (2012). Establishing and sustaining research partnerships in Africa: a case study of the UK-Africa Academic Partnership on Chronic Disease. Glob Health.

[16] Lown B, Banerjee A (2006). The developing world in the New England Journal of Medicine. Glob Health.

[17] Walsh A, Mulambia C, Brugha R, Hanefeld J (2012). The problem is ours, it is not CRAIDS’. Evaluating sustainability of Community Based Organisations for HIV/AIDS in a rural district in Zambia. Glob Health.

[18] Crisp N (2014). Mutual learning and reverse innovation-where next. Glob Health.

[19] Ebi KL (2008). Adaptation costs for climate change-related cases of diarrhoeal disease, malnutrition, and malaria in 2030. Glob Health.

[20] Hoy D, Roth A, Lepers C, Durham J, Bell J (2014). Adapting to the health impacts of climate change in a sustainable manner. Glob Health.

[21] Cohen JC (2007). Canada’s implementation of the Paragraph 6 Decision: is it sustainable public policy?. Glob Health.

[22] Chakma J, Masum H, Perampaladas K (2011). Indian vaccine innovation: the case of Shantha Biotechnics. Glob Health.

[23] Wilson KR, Kohler JC, Ovtcharenko N (2012). The make or buy debate: considering the limitations of domestic production in Tanzania. Glob Health.

[24] Kanavos P, Vandoros S, Garcia P (2009). Benefits of global partnerships to facilitate access to medicines in developing countries: a multi-country analysis of patients and patient outcomes in GIPAP. Glob Health.

[25] Lexchin J (2013). Canada and access to medicines in developing countries: intellectual property rights first. Glob Health.

[26] Scheffler RM, Pathania V (2005). Medicines and vaccines for the world’s poorest: is there any prospect for public-private cooperation?. Glob Health.

[27] Zurovac D, Otieno G, Kigen S, Mbithi AM (2013). Ownership and use of mobile phones among health workers, caregivers of sick children and adult patients in Kenya: cross-sectional national survey. Glob Health.

[28] Lang A (2014). Government capacities and stakeholders: what facilitates ehealth legislation?. Glob Health.

[29] Mackey TK, Liang BA (2013). Pharmaceutical digital marketing and governance: illicit actors and challenges to global patient safety and public health. Glob Health.

[30] Moore L, Chersich MF, Steen R, Reza-Paul S, Dhana A, Vuylsteke B (2014). Community empowerment and involvement of female sex workers in targeted sexual and reproductive health interventions in Africa: a systematic review. Glob Health.

[31] Aantjes C, Quinlan T, Bunders J (2014). Integration of community home based care programmes within national primary health care revitalisation strategies in Ethiopia, Malawi, South-Africa and Zambia: a comparative assessment. Glob Health.

[32] Tashobya C, da Silveira V, Ssengooba F, Nabyonga-Orem J, Macq J, Criel B (2014). Health systems performance assessment in low-income countries: learning from international experiences. Glob Health.

[33] Estimé MS, Lutz B, Strobel F (2014). Trade as a structural driver of dietary risk factors for noncommunicable diseases in the Pacific: an analysis of household income and expenditure survey data. Glob Health.

[34] Basu S, Stuckler D, Gonsalves G, Lurie M (2009). The production of consumption: addressing the impact of mineral mining on tuberculosis in southern Africa. Glob Health.

[35] Bandara M, Ananda M, Wickramage K, Berger E, Agampodi S (2014). Globalization of leptospirosis through travel and migration. Glob Health.

[36] Veenstra N, Whiteside A, Lalloo D, Gibbs A (2010). Unplanned antiretroviral treatment interruptions in southern Africa: how should we be managing these. Glob Health.

[37] Hirschhorn LR, Talbot JR, Irwin AC, May MA, Dhavan N, Shady R (2013). From scaling up to sustainability in HIV: potential lessons for moving forward. Glob Health.

[38] Sandoval C, Cáceres CF (2013). Influence of health rights discourses and community organizing on equitable access to health: the case of HIV, tuberculosis and cancer in Peru. Glob Health.

[39] Wibulpolprasert S, Chokevivat V, Oh C (2011). Government use licenses in Thailand: the power of evidence, civil movement and political leadership. Glob Health.

[40] Reynolds L, McKee M (2010). Organised crime and the efforts to combat it: a concern for public health. Glob Health.

[41] Silva C (2012). Non-communicable diseases in Mozambique: risk factors, burden, response and outcomes to date. Glob Health.

[42] Echouffo JB (2011). Chronic non-communicable diseases in Cameroon-burden, determinants and current policies. Glob Health.

[43] Kolling M, Winkley K, Deden Von M (2010). “For someone who’s rich, it’s not a problem” Insights from Tanzania on diabetes health-seeking and medical pluralism among Dar es Salaam’s urban poor. Glob Health.

[44] MacLachlan M (2009). Rethinking global health research: towards integrative expertise. Glob Health.

[45] D’Agostino M, Martin G (2009). The bioscience revolution & the biological weapons threat: levers & interventions. Glob Health.

[46] Bruen C, Brugha R, Kageni A, Wafula F (2014). A concept in flux: questioning accountability in the context of global health cooperation. Glob Health.

[47] Yach D (2008). The role of business in addressing the long-term implications of the current food crisis. Glob Health.

[48] Matheka DM, Nderitu J, Mutonga D, Otiti MI (2014). Open access: academic publishing and its implications for knowledge equity in Kenya. Glob Health.

[49] Jones F, Knights D, Sinclair W, Baraitser P (2013). Do health partnerships with organisations in lower income countries benefit the UK partner? A review of the literature. Glob Health.

[50] Von Schirnding Y (2005). The world summit on sustainable development: reaffirming the centrality of health. Glob Health.

[51] Hill PS, Dodd R, Brown S, Haffeld J (2012). Development co-operation for health: reviewing a dynamic concept in a complex global aid environment. Glob Health.

[52] Bai L, Morton LC, Liu Q (2013). Climate change and mosquito-borne diseases in China: a review. Glob Health.

[53] Bowen KJ, Friel S (2012). Climate change adaptation: Where does global health fit in the agenda. Glob Health.

[54] Bertoldi AD, Helfer AP, Camargo AL (2012). Is the Brazilian pharmaceutical policy ensuring population access to essential medicines. Glob Health.

[55] Pocock NS, Phua KH (2011). Medical tourism and policy implications for health systems: a conceptual framework from a comparative study of Thailand, Singapore and Malaysia. Glob Health.

[56] Bloom G, Henson S, Peters DH (2014). Innovation in regulation of rapidly changing health markets. Glob Health.

[57] Harris B, Eyles J, Penn L (2014). Adverse or acceptable: negotiating access to a post-apartheid health care contract. Glob Health.

[58] Naidoo R, Johnson K (2013). Community-based conservation reduces sexual risk factors for HIV among men. Glob Health.

[59] Reeves A, Basu S, McKee M, Meissner C (2013). Does investment in the health sector promote or inhibit economic growth. Glob Health.

[60] Maru D, Andrews J, Schwarz D (2012). Crossing the quality chasm in resource-limited settings. Glob Health.

[61] Soewondo P, Ferrario A, Tahapary DL (2013). Challenges in diabetes management in Indonesia: a literature review. Glob Health.

[62] Farrington C, Aristidou A, Ruggeri K (2014). mHealth and global mental health: still waiting for the mH2 wedding?. Glob Health.

[63] Kishore SP, Bitton A, Cravioto A, Yach D (2010). Enabling access to new WHO essential medicines: the case for nicotine replacement therapies. Glob Health.

[64] McKee M, Haines A, Ebrahim S, Lamptey P (2014). Towards a comprehensive global approach to prevention and control of NCDs. Glob Health.

[65] Silva A (2013). Determinants of unequal HIV care access among people living with HIV in Peru. Glob Health.

[66] Spicer N, Bogdan D, Harmer A, Murzalieva G, Semigina T (2011). ‘It’s risky to walk in the city with syringes’: understanding access to HIV/AIDS services for injecting drug users in the former Soviet Union countries of Ukraine and Kyrgyzstan. Glob Health.

[67] Skovdal M, Ogutu VO (2009). “I washed and fed my mother before going to school”: understanding the psychosocial well-being of children providing chronic care for adults affected by HIV/AIDS in Western Kenya. Glob Health.

[68] Brolan CE, Lee S, Kim D, Hill PS (2014). Back to the future: what would the post-2015 global development goals look like if we replicated methods used to construct the Millennium Development Goals?. Glob Health.

